# Diagnostic Methods for Non-Falciparum Malaria

**DOI:** 10.3389/fcimb.2021.681063

**Published:** 2021-06-17

**Authors:** Alba Marina Gimenez, Rodolfo F. Marques, Matías Regiart, Daniel Youssef Bargieri

**Affiliations:** ^1^ Department of Parasitology, Institute of Biomedical Sciences, University of São Paulo, São Paulo, Brazil; ^2^ Department of Clinical and Toxicological Analyses, School of Pharmaceutical Sciences, University of São Paulo, São Paulo, Brazil; ^3^ Department of Fundamental Chemistry, Institute of Chemistry, University of São Paulo, São Paulo, Brazil

**Keywords:** non-falciparum malaria, *Plasmodium* sp, PoC (point of care), differential diagnosis, malaria control and elimination

## Abstract

Malaria is a serious public health problem that affects mostly the poorest countries in the world, killing more than 400,000 people per year, mainly children under 5 years old. Among the control and prevention strategies, the differential diagnosis of the *Plasmodium*–infecting species is an important factor for selecting a treatment and, consequently, for preventing the spread of the disease. One of the main difficulties for the detection of a specific *Plasmodium* sp is that most of the existing methods for malaria diagnosis focus on detecting *P. falciparum*. Thus, in many cases, the diagnostic methods neglect the other non-falciparum species and underestimate their prevalence and severity. Traditional methods for diagnosing malaria may present low specificity or sensitivity to non-falciparum spp. Therefore, there is high demand for new alternative methods able to differentiate *Plasmodium* species in a faster, cheaper and easier manner to execute. This review details the classical procedures and new perspectives of diagnostic methods for malaria non-falciparum differential detection and the possibilities of their application in different circumstances.

## Introduction

Human malaria is an infectious disease of great relevance in tropical and subtropical regions worldwide. This disease threatens more than 40% of the world population, causing 229 million cases and 410,000 deaths per year (World Health Organization, 2020).

Malaria eradication has been a main goal of scientific and public health communities over the last century, which prompted the World Health Organization (WHO) to establish the Global Malaria Eradication Programme in the 1950s. After failed attempts, the focus shifted towards local control strategies, and in recent decades, huge efforts converged aiming at malaria elimination and future eradication. However, the prior objective of eliminating malaria in 35 new endemic countries from 2015 to 2030 seems to be unreachable (Brew et al., 2020). The Lancet Commission on Malaria Eradication considered that malaria eradication by 2050 is a feasible and affordable goal (Feachem et al., 2019). Nevertheless, in the meantime, this disease remains a major global health problem. Cases of imported malaria in non-endemic regions such as European countries and the US can cause secondary local transmission, contribute to the spread of drug resistance and threaten long-term eradication goals (Tatem et al., 2017).

Malaria is caused by protozoan parasites belonging to the genus *Plasmodium*. Among the species that infect humans, *P. falciparum* (*Pf*) is the main cause of the severe form of the disease and death, accounting for 99.7% of infections in Sub-Saharan Africa. *P. vivax* (*Pv*), on the other hand, is the species most widely distributed and prevalent in most malarial areas outside of Africa, and is responsible for 75% of infections in the Americas (World Health Organization, 2020). Other species causing human malaria, such as *P. malariae (Pm), P. ovale (Po) curtisi* and *Po wallikeri*, are also widely dispersed, with less prevalence than *Pf* and *Pv* in most malaria-endemic areas. However, the disease pathogenesis that these species cause is similar to *Pv*; and may lead to severe cases of malaria, sometimes even lethal (Mueller et al., 2007). Likewise, *P. knowlesi (Pk)*, which is a non-human primate malaria parasite, causes zoonotic human infections in Southeast Asia with morbidity similar to *Pf* (Ahmed and Cox-Singh, 2015). Early diagnosis of malaria is one of the most important forms of control, since it allows for rapid treatment and prevents further progression of transmission. However, the symptoms caused by malaria are often indistinguishable from symptoms caused by diseases such as viral hepatitis, dengue and leptospirosis, among others, which complicates the diagnosis (Gadia et al., 2017). To overcome this problem, several diagnostic methods were developed to detect mainly *Pf* infections and its by-products. However, diagnostic tools currently available frequently neglect non-falciparum species or do not discriminate among them.

Besides the lack of scientific knowledge about the biology of other *Plasmodium* species when compared to *Pf* or even *Pv*, research groups studying non-falciparum malaria must overcome obstacles related to the biology of these parasites. This is a factor that significantly impacts the success in developing new strategies to improve the specific and accurate detection of these species. First, parasitemia is typically very low (even microscopically undetectable) in *Pv*- and *Pm*-infected individuals. Two factors contribute for this situation: at the end of the hepatic cycle, each ruptured hepatocyte releases approximately 2,000 merozoites when the infection is due to *Pm* and 10,000 when due to *Pv*, whereas ~40,000 merozoites/hepatocyte are released when the infection is due to *Pf*. Moreover, *Pm* only invades old red blood cells (0.1% of the total) and *Pv/Po* preferentially invade young red blood cells (1% of the total), whereas *Pf* invades red blood cells in any developmental stage (Kerlin and Gatton, 2013).

Second, *Pv* and *Po* exhibit slow development of some of its sporozoites, forming hypnozoites, latent forms of the parasite responsible for disease relapses which can contribute up to 80% of all infections in the blood stage and, if not treated properly, can cause new infections within months and even years after the primary infection (White, 2011; Betuela et al., 2012). To date, primaquine is the only drug approved for preventing relapses of *Pv-* and *Po-*infections. Nevertheless, this drug is incompatible with glucose-6-phosphate-dehydrogenase (G6PD) deficiency, and therefore a careful evaluation must be made before prescription (Hanboonkunupakarn and White, 2020). There are other challenges regarding hypnozoites, such as the difficulty of detection, since these dormant forms express a smaller set of genes than replicative schizonts in the liver (Voorberg-van der Wel et al., 2017). For these reasons, diagnostic tools currently available are unable to detect hypnozoites. In the last years, efforts to identify markers for distinguishing between dormant forms and replicating parasites in the liver gave promising results (Gualdron-Lopez et al., 2018; Schafer et al., 2018) further research is necessary to effectively detect patients harboring hypnozoites in their liver.

Finally, relapses and submicroscopic infections by non-falciparum species are frequently asymptomatic, which makes their detection even more difficult because, as there are no symptoms, infected people do not seek out a health center. Those infections produce gametocytes and are likely to substantially contribute to maintaining non-falciparum malaria reemergence and transmission, even in low-endemicity areas, such as most of the Amazon Basin (da Silva-Nunes et al., 2012; Antonelli et al., 2020) and the Peruvian Amazon (Carrasco-Escobar et al., 2017; Rovira-Vallbona et al., 2017).

Thus, the lack of analytical sensing tools that allow for early and accurate detection in low-parasitemia circumstances, and the failure to diagnose the right *Plasmodium* species, are important factors that contribute to the malaria persistence and increase the parasite’s resistance to antimalarials, given that rational drug use is dependent on prompt and accurate malaria diagnosis (Landier et al., 2016). In particular, early detection and *Plasmodium* sp differentiation became of extreme importance after the emergence of resistance to chloroquine, and thus, specific therapeutic schemes were formulated to avoid further increase of antimalarial drug resistance (Buyon et al., 2021; Patel et al., 2021). In general terms, chloroquine is the preferred agent if the infection is considered uncomplicated; thus, is the treatment suggested for most non-falciparum infections. For *Pf*-infections a variety of antimalarial agents is used; frequently quinones and artemisinin derivatives. For *Pv*- and *Po*- infections, as mentioned, an additional treatment with primaquine is necessary to avoid future relapses (Hanboonkunupakarn and White, 2020; Hill et al., 2021).

The next sections of this review present our current knowledge about the efforts seeking to implement, improve or develop diagnostic techniques for the detection of non-falciparum malaria. These include classic diagnostic methods currently applied in the field, such as microscopy and rapid diagnostic test (RDT); methods requiring laboratory techniques such as nucleic acid detection and immunological assays; and new platforms using high throughput and potentially in field applicable devices such as Loop-mediated isothermal amplification (LAMP) and biosensing approaches. We also discuss notable perspectives that can be developed for the rational design of new and effective diagnostic tools.

## Field Diagnostic Methods

The diagnosis of malaria must take into account three related criteria: clinical, epidemiological and laboratory. The first is based on the analysis of the initial symptoms of the disease such as nausea, headache, vomiting, profuse sweating and myalgia. These signs are common to malaria and other febrile illnesses, therefore an epidemiological criterion is needed for a differential diagnosis. With the lack of specific clinical parameters to confirm the infection, laboratory methods for the accurate diagnosis of malaria in endemic areas are needed.

In **Table 1**, a summary of the different diagnostic methods covered in this review is presented. The applicability of each method depends on the different situations in which the diagnosis occurs. For point-of-care (PoC) detections, the rapid and accurate diagnosis of individuals seeking a health center, currently there are only two techniques widely used: microscopy and RDTs. Their strengths and weaknesses as diagnostic methods are discussed in this section.

**Table 1:** Summary of diagnostic methods for non-falciparum (nf) malaria.

Classification	Method	Sensitivity (a)	Advantages or Strengths	Disadvantages or Weaknesses
		Specificity for nf spp (b)		
**Field diagnosis**	Microscopy (Section 2a)	a. LOD ~50-200 parasites/µL	Gold-standard method for malaria diagnosis in field.	Unable to detect sub-microscopic infections.
		b. High in single infections	Low cost	Requires well-trained technicians.
			PoC detection	Eventual spp misidentification.
				Under-diagnosis of minority spp in mixed infections.
	RDTs (Section 2b)	a. Expected: 75% at 200 parasites/μL	Rapid (~20 minutes)	Unable to detect sub-microscopic infections.
		b. not specific for nf spp except Types 5-6 for Pv (see **Table 2**)	Low cost – independent of equipment	Unable to differentiate among nf spp.
			Requires minimal training	Low sensitivity for nf spp in field (frequently lower than expected).
			PoC detection	
**Laboratory diagnosis**	Immunological methods (Section 3a)	a. Commercial ELISA kits: 95% to detect clinical malaria.	Useful for epidemiological surveys seeking to study malaria prevalence.	Requires trained personnel and laboratory equipment.
		b. Commercial ELISA kits: not specific for nf spp.	Promising results on detection of active infections could be useful to develop new RDTs or biosensors.	In-house assays were validated in field using relatively low numbers of samples and require further validation to determine sensitivity and spp specificity.
	Detection of iRBCs (Section 3b)	–	Promising results that require further validation.	Requires trained personnel and laboratory equipment.
				High cost of required equipment.
	PCR (Section 3c)	a. LOD 0.2-5 parasites/µL (blood)	Gold-standard method for detecting sub-microscopic infections.	Requires trained personnel and laboratory equipment.
		b. High (~85-100%, compared to microscopy).	Quantitative determination (qPCR).	High cost of reagents and equipment.
			Most assays were able to differentiate among nf spp.	Even though several assays were validated with samples from endemic areas, this method remains mostly used only for research purposes.
**New potential PoC diagnosis**	Biosensors (Section 4a)	a. Highly variable.	Analytical performance well documented. Promising results that require further validation.	Currently requires trained personnel and laboratory equipment.
		b. Mostly specific for Pf and Pv determination.		These methods were not yet validated in field.
	“Lab-on-chip” or LAMP-based methods (Section 4b)	a. High, ~95-100% compared to PCR.	Requires less training and equipment than PCR, while obtaining similar results.	Prone to contamination (requires high care in sample and reagents manipulation).
		b. High, ~85-100% depending on the assays and the comparator.	Commercial kits with high sensitivity available.	Commercial kits currently available do not discriminate among nf spp.
			Potentially PoC.	

In most rural settings where malaria is endemic, the structure necessary for molecular diagnostic techniques is not available. In these cases, samples are sent to specific laboratories for molecular analysis and/or seroepidemiological studies. Because of the time window between sample collection and analysis, these tests are not routinely used to inform correct treatment, but help establish the epidemiological criteria required to complement the differential diagnosis.

### Microscopy

The microscopic observation of blood samples has advantageous characteristics for diagnosing malaria, such as low cost, portability, specificity and sensitivity. For these reasons, microscopy remains the gold standard for malaria detection in the field.

This technique is based on the collection of peripheral blood from patients with suspected malaria infection for the preparation of slides. The diagnosis is made by observing thick drop and thin blood films. Frequently, the thick drop is the method of choice for malaria diagnosis, because a greater amount of blood cells can be observed in a relatively small area, increasing the probability of finding infected red blood cells (iRBC). Slides are stained with Giemsa solution or other specific dyes and examined with a 100X oil immersion objective in optical microscopes. This method enables differentiation of the parasite at a species level, and also provides a count of parasites per field, therefore being qualitative and quantitative (Warhurst, 1990). It is worth mentioning that the identification of the infecting *Plasmodium* species is easier using the thin blood film, as it allows a better study of the parasite’s morphology and the characteristic changes of the parasitized erythrocyte. The microscopic examination allows for the detection of up to 5-10 parasites/µL of blood, however, this limit is dependent on the previous training and experience level of the microscopist for interpreting the test. In the field, the limit of detection (LOD) for this method is approximately 50-200 parasites/µL. The total time for applying this exam is typically about 60 minutes (Payne, 1988).

Although microscopy is still the most used method for diagnosing malaria (Berzosa et al., 2018), many biases are involved, such as: the technical skills in preparing the slide; lysis of red blood cells and consequent changes in parasite morphology (leading to errors in identifying species); optical quality and microscope illumination; competence and care by the microscopist, and, eventually, the level of parasitemia (Hanscheid, 2003; World Health Organization, 2016). An important point is that due to the LOD, individuals with low-parasitemic (submicroscopic) infections, mostly asymptomatics, will remain undiagnosed and untreated, enabling the transmission cycle to continue in the community (Berzosa et al., 2018). Also, the differentiation among *Plasmodium* species is a key point for effective treatment and, consequently, for the success in malaria control and elimination. In this context, it is not uncommon for failures to occur in the microscopical identification of *Plasmodium* species, even when performed by well-trained microscopists. The misidentification is more prevalent in non-falciparum malaria endemic areas (Diallo et al., 2018). The parasite density in infections by non-falciparum *Plasmodium* species is usually low compared to *Pf* (Obare et al., 2013). Moreover, the microscopist’ performance in correctly detecting malaria-infected samples depends on the *Plasmodium* spp. For instance, in contrast to their good performance in diagnosing *Pf*, microscopists participating in proficiency tests have commonly more difficulty diagnosing *Pm*, *Po*, and *Pv* (Edson et al., 2010). This difficulty leads to under-diagnosis of non-falciparum malaria, especially when present in mixed infections with *Pf* as the predominant specie. In these cases, treatment will normally be against *Pf* (most probably artemisinin); while co-infecting *Pv*- and *Po*- would also require treatment with primaquine in order to avoid future relapses (Kakkilaya, https://www.malariasite.com/ treatment-of-malaria/).

Since many countries only have microscopy or rapid tests as diagnostic tools, tests evaluating the performance of laboratory technicians trained in malaria microscopy were carried out. The analysis showed that there were consistent failures in the identification of *Plasmodium* species, such as overlooked *Po*-infections with low parasite densities, *Pf*-infected samples misidentified as *Pm*, and *Po* slides misidentified as *Pv* (Edson et al., 2010; Diallo et al., 2018). Particularly, the misidentification of *Pf*-infected samples as *Pm*, *Po* or *Pv* could lead to administration of inefficient treatment (chloroquine instead of artemisinin), thus increasing the risk of severe malaria. Additionally, in endemic African countries misidentifications of *Plasmodium* spp have been reported, particularly for the non-falciparum species (Mukadi et al., 2016) and especially for those with low prevalence (Obare et al., 2013). These data indicate a tendency for clinical laboratories to neglect non-falciparum malaria due to their less severe clinical symptoms compared to falciparum malaria (Diallo et al., 2016).

It is necessary to point out that species determination and distinction, especially when different species share morphological similarities, are common failure points of microscopy as a diagnostic method. It is not uncommon for well-trained microscopists to have difficulty distinguishing early trophozoites of *Pv* from those of *Pf*, particularly when parasitemia is low. Thus, in general, microscopists tend to interpret slides that are difficult to read (either because of morphology, artifacts or low parasitemia) as *Pf* rather than *Pv* (Alemu et al., 2014). As previously mentioned, this misidentification would lead to treatment choices that would not prevent relapses. On the other hand, in *Pv*-endemic areas, mixed *Pv* + *Pf* infections may be misdiagnosed as *Pv*-monoinfections; this may also lead to an inefficient treatment of *Pf* malaria with chloroquine (Ehtesham et al., 2015).

*Pv* also shares morphological and biological resemblances with *Po*, such as the similar tertian periodicity and the occurrence of relapses. For these reasons, *Po* is easily and commonly misidentified as *Pv* in routine diagnosis. Moreover, differentiations between *Po* and *Pv* can be even more challenging in low-parasitemic smears (Chavatte et al., 2015; Kotepui et al., 2020c). This misidentification would not have such high impact as others, since the recommended treatment is similar in both cases (Kakkilaya, https://www.malariasite.com/ treatment-of-malaria/).

There is also the difficulty in distinguishing *Pm* from other *Plasmodium* species using a microscopic method (Rahman et al., 2010; Obare et al., 2013). Thus, its prevalence is miscalculated, which may even result in severe complications (Kotepui et al., 2020b). In addition, *Pm* infections can easily be missed when microscopy is used, and patients are often treated for a bacterial infection. The incorrect treatment may even lead to kidney injury (Badiane et al., 2014). Also, the detection of *Pm* by the microscopy method in patients with mixed *Pm* + *Pf* infections in endemic areas where *Pf* predominates is difficult. These co-infections are frequent in areas of malaria endemicity in Africa (Collins and Jeffery, 2007).

Mixed infections, mostly misdiagnosed, are also frequent in co-endemic areas for *Pf*, *Pv* and *Pk* (Barber et al., 2013). Those misdiagnoses are also attributed to similar morphologies. For example, the early trophozoites of *Pk* are identical to those of *Pf*, with double chromatin dots, multiple infections per erythrocyte, and no enlargement of infected erythrocytes (Singh et al., 2004; Singh and Daneshvar, 2013). Both (*Pf* and *Pk*) infections are potentially life-threatening, therefore appropriate, accurate and early *Pk*-infections identification is crucial to administer the proper treatment, even saving lives (Lee et al., 2013). The most concerning misidentification of human-infecting *Plasmodium* spp is *Pk*-infections identified as more benign *Pm*-infections. The late *Pk* trophozoites (band-form) and schizonts resemble those of *Pm* and, therefore, cannot be reliably and accurately differentiated (Singh and Daneshvar, 2013; Kotepui et al., 2020b). This misidentification has been associated with the failure to diagnose severe malaria, resulting in fatal outcomes. For these reasons, in *Pk*-endemic areas, microscopic diagnosis of *Pm* should be reported and treated as *Pk* to reduce case-fatality rates (World Health Organization, 2017).

All of these failures occur more frequently in cases of imported malaria, especially those occurring in non-endemic areas since the laboratory routine is not as intense as in endemic areas. This can decrease accuracy in the efficiency of species differentiation, consequently contributing to failures in diagnosis and treatment (Moore et al., 1994; Kain et al., 1998).

In addition to these factors, accurate diagnosis of malaria through microscopy is difficult in many circumstances and countries, either because of the precariousness of health services, or due to the population’s difficulty in accessing diagnostic centers. For all of these reasons, over the years more reliable, practical, sensitive and faster methods to diagnose malaria have been developed. Nevertheless, to date, microscopy remains the gold standard for malaria diagnosis of symptomatic individuals. Most studies comparing accuracy or sensitivity, which will be addressed in the following sections, use microscopy as the reference method.

### Rapid Diagnostic Tests (RDT)

Immunochromatographic tests for malaria diagnosis, widely known as rapid diagnostic tests (RDT), consist of antibodies against *Plasmodium-*specific antigens immobilized on nitrocellulose membranes (capture antibody) and bound to gold particles or other visually detectable marker (labeled antibody in mobile phase). In these devices, the liquid mobile phase contains blood from people with suspected malaria infection. If infected, the migration of the complex “antigen - labeled antibody” present in the mobile phase results in a new immobilized complex “capture antibody – antigen - labeled antibody”. This binding allows the visualization of coloured lines formed by the immobilized antigen-antibody complexes (Kakkilaya, 2003).

Generally speaking, RDTs enable simple, effective malaria diagnoses that can be performed rapidly outside the laboratory, in PoC applications. After several years without guidance or formal evaluation, the World Health Organization (WHO) in collaboration with other Malaria Programs, performed quality controls and lot testing of RDT products (Cunningham et al., 2019). From 2009 to 2013, the WHO Malaria Policy Advisory Committee established the minimum recommended procurement criteria: any RDT should be fast and independent of equipment, accessible to those in need and require minimal training. As for sensitivity and specificity of these tests, the WHO recommended that the panel detection score against *Pf* and *Pv* samples should be at least 75% at 200 parasites/μL density, with a false positive rate of < 10% and invalid rate of < 5% allowed in all transmission settings (Cunningham et al., 2019). Furthermore, the WHO, together with the Foundation for Innovative New Diagnostics (FIND), coordinated the WHO Malaria RDT Product Testing Programme, aimed at ensuring the quality of the tests. To date, eight rounds of testing have been completed since 2008 (World Health Organization, 2018), and information about RDTs detection rates, specificity and accuracy is available at the WHO webpage (WHO, https://www.who.int/malaria/areas/diagnosis/rapid_diagnostictests/en/).

Currently, there are several RDTs available on the market. The most commonly used antigens for malaria diagnosis are *Pf*-specific histidine-rich protein II (PfHRP2) for *Pf*; *Plasmodium* pan-specific lactate dehydrogenase (pLDH) and pan-malarial Aldolase for all *Plasmodium* species that infect humans (Krampa et al., 2017). Despite their low sensitivity and lack of specificity for non-falciparum species, RDTs are still the most used technique for malaria detection in resource-limited endemic settings, since they require no electricity or laboratory infrastructure and yield results within 15-20 minutes. A disadvantage is their sensitivity to detect non-falciparum spp, which overall is low and even lower when excluding also *Pv*. Another important disadvantage is the emergence of PfHRP2/3-deleted parasites in several countries where malaria is endemic. HRP2-based RDTs are widely used, being a selective pressure on the parasite population to drive the spread of PfHRP2/3-deleted strains (Watson et al., 2017). Thus, the false-negative rate in symptomatic patients can be alarming. The performance of PfLDH-based RDTs is good in high-density *Pf* samples lacking PfHRP2, but highly variable at lower densities (Gatton et al., 2020).

As depicted in **Table 2**, existing tests that are able to differentially detect *Pf* and non-falciparum infections are either Type 2 (detect PfHRP2 and panmalarial Aldolase), Type 3 (detect PfHRP2 and pLDH), Type 4 (detect *Pf*-specific LDH and pLDH), Type 5 (detect *Pf-* and *Pv*-specific LDH) or Type 6 (detect PfHRP2, *Pv*-specific LDH and pLDH). The best-performing RDTs available for the specific diagnosis of *Pv* (Type 6) reached a sensitivity of 95% compared to microscopy. For PoC detection, such sensitivity is still below what is necessary for the frequent submicroscopic parasitemia scenarios. Moreover, the most-frequently used RDTs in the field (Types 2, 3 and 4) present sensitivity of 75-87% for *Pv* microscopically-confirmed infections (Abba et al., 2014). To date, there are no RDTs specifically designed for *Po, Pm* or *Pk* detection.

**Table 2:** Rapid diagnostic test (RDT) types for malaria detection.

RDT types	Antigens detected	Expected Spp differential detection	Interpretation of positive signal ^(#)^
**Type 1**	Line 1: PfHRP2	Pf	L1 = Pf
**Type 2**	Line 1: PfHRP2	Pf/*Plasmodium* spp	L1 + L2 = Pf
	Line 2: pan-Aldolase^(*)^		L2 = nf spp
**Type 3**	Line 1: PfHRP2	Pf/*Plasmodium* spp	L1 + L2 = Pf
	Line 2: pan-LDH^(*)^		L2 = nf spp
**Type 4**	Line 1: PfLDH	Pf/*Plasmodium* spp	L1 + L2 = Pf
	Line 2: pan-LDH^(*)^		L2 = nf spp
**Type 5**	Line 1: PfLDH	Pf/Pv	L1 = Pf
	Line 2: PvLDH		L2= Pv
			L1 + L2 = mixed Pf + Pv
**Type 6**	Line 1: PfHRP2	Pf/Pv/*Plasmodium* spp	L1 + L3 = Pf
	Line 2: PvLDH		L2 + L3 = Pv
	Line 3: pan-LDH^(*)^		L1 + L2 + L3 = mixed Pf + Pv
			L3 = nf, nv spp
**Type 7**	Line 1: pan-Aldolase^(*)^	*Plasmodium* spp	L1 = *Plasmodium* spp

^(#)^RDT Control line (L0) is assumed to be positive in all cases.

^(*)^Positive signal in pan-Plasmodium antigens lines could represent or not mixed infections. nf, non-falciparum; nv, non-vivax.

The sensitivity of existing RDTs for detection of those species was tested in several works, using blood samples from monoinfected-patients. In a systematic review, Yerlikaya et al. (2018) determined that for *Po* and *Pm* detection, results were inconclusive due to the small number of samples tested (ranging from 0% to 100% sensitivity); however, the sensitivity was very low in most studies. For *Pk* detection as non-falciparum malaria, a 48% average sensitivity was observed for type 3 RDTs (24% for Type 2, 12% for Type 4) (Yerlikaya et al., 2018).

The misdiagnosis observed could be due to several factors: the monoclonal antibody used to detect *Pf* (HRP2) cross-reacts with *Pk*; therefore the RDT could identify the infection as a single or mixed *Pf* infection (Amir et al., 2018). As for Type 6 RDTs, sensitivity for *Pk* was 2% (Yerlikaya et al., 2018). Those RDTs are specific for *Pv* lactate dehydrogenase, and can mistakenly detect *Pk* as *Pv*. Moreover, those kits fail to detect *Pk* infection in patients with low parasitemia (Singh and Daneshvar, 2013).

In a recent work, the performance of three pLDH-based RDTs for *Po wallikeri* and *Po curtisi* detection was evaluated. The Wondfo diagnostic kit for malaria (Pf/Pan) was considered the best performing, with 70% sensitivity vs. 55% sensitivity for the CareStart Malaria pLDH (Pan) and 18% sensitivity for the SD BIOLINE Malaria Ag (Pf/Pan). When analyzing the specific sensitivity towards each subspecies, the Wondfo kit demonstrated a similar sensitivity to *Po wallikeri* and *Po curtisi* (67-73%, respectively), while the CareStart kit showed better sensitivity to *Po wallikeri* (75% vs. 36.5% to *Po curtisi*) and the SD BIOLINE was only able to detect *Po wallikeri* (37.5% vs. 0% to *Po curtisi*). Moreover, the overall detection ratio of all three RDTs decreased with parasite density. Even when the parasitemia was higher than 5,000 parasites/μL, these RDT didn’t reach 80% sensitivity (Tang et al., 2019).

Finally, there is discrepancy in studies testing the performance of currently available RDTs in the detection of mixed *Pf*/non-falciparum infections (Kotepui et al., 2020a). In general, Type 3 Malaria RDTs could detect a higher proportion of *Plasmodium* mixed infections than RDTs targeting PfHRP2/*Pv*-specific LDH; however, Type 3 RDTs also yield frequent false-positives (Berzosa et al., 2018). To date, any suspected case of mixed infection must be confirmed or discarded by microscopy and PCR standards in order to administer the proper treatment.

## Laboratory Diagnostic Methods

As previously mentioned, laboratory diagnosis of malaria is rarely performed at point of care, with the probable exception of returning travelers from endemic areas and health centers established in specific settings. In the following section, laboratory diagnostic methods which require trained personnel and laboratory equipment will be addressed. Immunological methods are mostly used for epidemiological studies, while molecular detection by PCR is the gold-standard method for detecting sub-microscopic infections (see **Table 1**).

Several comparative studies discussed in the following sections highlight the challenge of comparing different diagnostic methods and assays, as the reported units regarding sensitivity and detection limits may be different. We attempted to maintain the use of units reported in each study. In general terms, as described for RDTs, in immunological assays the reported sensitivity is referred to percentages of microscopically-confirmed infections. Regarding detection limits reported in molecular methods, although not accurate for some parasite forms, in general the most used units (parasites/μL and iRBC/μL) are equivalent. In these methods, the usual comparator for sensitivity results is nested-PCR. Finally, some techniques and assays, mostly not validated in the field, reported the analytical performance, making the comparison to other results even more difficult.

### Immunological Methods

Serological diagnosis of malaria is based on the detection of antibodies against blood-stage parasites present in the serum of malaria-infected patients. The immunological diagnostic methods more frequently used are Immunofluorescence antibody assay (IFA) and Enzyme-linked immunosorbent assay (ELISA). These immunological assays were classically used for surveillance and diagnosis of previous malaria exposition. However, there are ELISA assays based on detection of antibodies against specific antigens proposed as diagnostic tools for recent or active infections, which will be discussed in this section.

IFA was considered the gold-standard method for malaria serology testing, particularly because it was useful in epidemiological surveys and screening of potential blood donors or diagnosis of recent infections in non-immune patients (Tangpukdee et al., 2009). The method consists of parasite blood-stage forms immobilized on microscope slides as antigens to antibodies present in infected samples. Thus, serum samples from patients, and a secondary antibody labeled with fluorophores, are subsequently incubated with the slides. If the sample contains anti-*Plasmodium* antibodies, the formed complexes are observed in a fluorescence microscope.

Although IFA demonstrated high sensitivity, further validation assays revealed an alarming rate of false-positivity of malaria diagnosis in non-immune travelers (Miranda et al., 2008). Also, the methodology is time-consuming, it cannot be automated and requires fluorescence microscopy and trained technicians; particularly for serum samples with low antibody titers (Tangpukdee et al., 2009). Moreover, it requires slides containing specific antigens, prepared with blood samples obtained from patients with high parasitemia (De Carvalho et al., 1992) or *in vitro*-cultured parasites. Those difficulties, and the equal or higher sensitivity and specificity of Enzyme Immunoassays (Doderer et al., 2007; She et al., 2007), prompted the development of ELISA as a serological method to detect malaria.

The ELISA method used for serological diagnosis follows the same rationale as the IFA, detection of antibodies against malarial antigens potentially present in serum samples, using a labeled secondary antibody. The main differences are the use of 96-well plates instead of slides, allowing the simultaneous testing of multiples samples; the use of recombinant proteins as antigens for immobilization rather than whole parasites; and the method of result visualization. Secondary antibodies for ELISA are commonly labeled with enzymes that react with colorimetric substrates. Therefore, the antigen-antibody complex formation can be measured using a spectrophotometer.

As occurring with IFA and RDTs, ELISA tests to detect malaria were first based on *Pf* antigen detection, with poor sensitivity against non-falciparum species (De Carvalho et al., 1992; She et al., 2007). In the last few decades, however, efforts to better understand the seroepidemiology of malaria-endemic areas as part of the Malaria Elimination Program have prompted the search for reliable targets for the specific detection of all *Plasmodium* spp.

Currently, there are commercial ELISA kits to detect antimalarial antibodies. The performance of five commercial kits (Bio-Rad/Trinity Biotech, Newbio, DiaPro, Cellabs and NovaTec), all detecting pan-*Plasmodium* spp anti-antibodies, was evaluated in a recent study (van den Hoogen et al., 2020). Specificity (*vs*. malaria unexposed donors or samples positive to *Toxoplasma*) was 96% except for the Cellabs kit (81-84%) and sensitivity ranged 90–95% except for the DiaPro kit (86%). There are also antigen detection ELISA kits, most of them for specific detection of PfHRP2, but also pan-malarial, such as Quantimal™ Ultrasensitive pLDH Malaria Ag CELISA (Cellabs), Malaria Antigen ELISA (apDia) and Malaria Ag ELISA kit (Creative Diagnostic), all of them for the detection of pan-*Plasmodium* pLDH. These commercial kits are regularly used by blood banks to discard possible malaria infections. Nonetheless, they do not discriminate among *Plasmodium* spp. Among non-falciparum species, *Pv* is the most studied target. Commercial ELISA kits (Genedia malaria antigen ELISA test and Genedia malaria antibody ELISA 2.0) were developed to detect *Pv*-specific LDH enzyme and antibodies, respectively. The antibody ELISA test was effective to detect antibodies against *Pv* in blood samples for screening (Nam et al., 2010) and the combined Genedia malaria antigen and antibody ELISA 2.0 tests demonstrated a clinical sensitivity of 98.3% (295/300) and a clinical specificity of 97.9% (714/729) (Kim et al., 2016). The tests did not assay the species specificity. Regarding in-house ELISA assays using these antigens, a monoclonal antibody against PvLDH (2CF5) was able to differentiate *Pf* from *Pv* in ELISA and IFA assays, suggesting that it might possess *Pv-*specificity. The limit of detection (LOD) for a pair of 2mAbs-linked sandwich ELISA was 31.3 ng/mL of the recombinant antigen (Linh et al., 2017). The specificity against other non-falciparum species remains to be determined.

The more evaluated target for serological studies is the merozoite surface protein 1 (MSP1), a glycoprotein expressed abundantly on the merozoite surface of all *Plasmodium* species. This protein contains highly immunogenic fragments that generate an immune response, activating the host immunity associated to protection (Holder, 2009; Beeson et al., 2016). Among some studies focusing on non-falciparum species, the application of MSP1 in immunoassays was studied early using *Pv* protein, PvMSP1, since antibodies generated by *Pv*-infected patients recognized specifically N-term and C-term domains of this protein (Soares et al., 1997). In another study, PvMSP1 was recognized by 62% of the sera from individuals recently exposed to *Pv* using indirect ELISA assay (Soares et al., 1999). A commercial ELISA kit to detect *Pf/Pv* infections (DiaMed ELISA malaria antibody test) was developed, based on a mixture of a total extract of cultured *Pf* and recombinant proteins PvMSP1+ *Pv* Circumsporozoite protein (PvCSP) (Doderer et al., 2007). The ELISA method was three times more sensitive than IFA for *Pv* infection (24 cases, 75% for ELISA and 25% for IFAT). However, when analyzing the performance of the kit use for blood screening, the sensitivity for proven *Pv* infections was 53%, compared to the results from microscopic examination and PCR testing (Oh et al., 2008). The specificity regarding other *Plasmodium* species was not assayed.

Among the immunogenic fragments of PvMSP1, the C-term portion was recognized by antibodies in a significant proportion of Brazilian individuals recently exposed to *Pv* (Soares et al., 1997) and *Pv*-infected or exposed individuals from Korea (Park et al., 2001; Lim et al., 2002). Thus, the 19-kDa fragment of PvMSP1, named PvMSP1_19_, was evaluated as an antigen for the specific serological detection of *Pv* malaria infection. The ELISA assay, exploring different recombinant protein constructions, all of them containing PvMSP1_19_ as the antigen, demonstrated high sensitivity (95% of total *Pv*-infected samples) when analyzing sera from naturally infected individuals from malaria-endemic areas. This assay also showed high specificity towards *Pv* detection when analyzing sera from healthy donors (100%) potentially exposed to *Pf* (95%) or infected with other infectious diseases (97.7%) (Rodrigues et al., 2003). Similar results were obtained in other works (Barbedo et al., 2007). Also, a multianalyte Dot-ELISA assay was developed for the determination of several infectious diseases using PvMSP1_19_ as a *Pv*-specific marker. This assay showed high sensitivity for anti-PvMSP1_19_ antibodies using samples from *Pv*-infected patients, regardless of whether it was their first malaria episode or not. The sensitivity of this assay was 90%; specificity towards *Pv* detection when analyzing sera from healthy donors or infected with other tropical diseases was 100%, and this assay was able to detect *Pv* in mixed infections with *Pf* (Coelho et al., 2007).

MSP1 genes from *Po* and *Pm* were also cloned and characterized (Birkenmeyer et al., 2010), and a new indirect ELISA test using the MSP1_19_ fragments from *Pf, Pv, Po* and *Pm* as antigens was developed. This assay was able to specifically detect all malaria patients with *Pf* (4/4) or *Pv* (8/8) infections, as did the commercial ELISA. However, the commercial ELISA detected antibodies in 0/2 and 5/8 individuals with *Pm* and *Po* infections, respectively, while the MSP1_19_-assays detected 100% of individuals with confirmed *Pm* or *Po* infections (Muerhoff et al., 2010). Regarding species specificity, another study by Priest et al. (2018) demonstrated that most antibody responses to the four MSP1_19_ antigens in a multiplexed serological assay were species-specific, indicative of previous infection. Specificity was 100% in experimentally infected chimpanzees, 11/12 human samples from the low transmission regions and 12/20 samples from the high transmission area (Priest et al., 2018).

The performance of these MSP1_19_-based ELISA assays is better when antigens are analyzed individually. For example, the recognition of PmMSP1_19_ was evaluated by ELISA using serum from Brazilian individuals diagnosed with malaria due to *Pm* (n = 16) in parallel with sera from patients with unrelated diseases (n = 21), or healthy donors (n = 15), reaching 100% sensitivity and specificity. On the other hand, sera from *Pv* or *Pf*-infected individuals did not react at all against recombinant PmMSP1_19_ protein (Elizardez et al., 2019). All three MSP1_19_ antigens (*Pf*, *Pv* and *Pm*) were recently used as bound-antigens in ELISA assays that were performed to evaluate the seroprevalence of these *Plasmodium* species in Suriname (Labadie-Bracho et al., 2020).

Differing from other non-falciparum species, MSP1 fragments from *Pk* were less evaluated as serological markers. In the few assays performed, a purified recombinant PkMSP1_33_ protein reacted with serum samples of patients infected with *Pk* by Western blot (100% sensitivity) and ELISA (80% sensitivity) assays. Most of the non-malarial infections (49/52) and healthy donor serum samples (65/65) did not react with PkMSP1_33_ protein, however, the assay failed to detect specifically *Pk* infections; other *Plasmodium* infections were equally detected (95-71%) using the recombinant with PkMSP1_33_ (Cheong et al., 2013). These results were similar to further studies using PkMSP1_19_ as antigen in serological detections by Western blot; PkMSP1_19_ reacted with 95.5% of the *Pk* samples (42/44), 81.6% of the non-*Pk* (*Pf, Pv, Po)* samples (31/38) and 0% of the healthy donor or non-malarial parasitic infections sera (133/133) (Sonaimuthu et al., 2015). A possible explanation for this cross-reactivity is the high level of amino acid identity (83%) between PvMSP1_19_ and PkMSP1_19_, which prompted the identification of a novel panel of *Pk* biomarkers of serological exposure (Herman et al., 2018). Through *in silico* tools, three cysteine proteases (SERA3Ag1, SERA3Ag2 and TSERA2Ag1) and the thrombospondin-related anonymous protein (TRAP) were identified, expressed and analyzed; of them, SERA3Ag2 had the highest prevalence among infected samples (64%) (Herman et al., 2018). In another study developing a panel of recombinant proteins from human−infective *Plasmodium* species for serological surveillance, MullerSienerth et al. (2020) predicted that *Pk* could be accurately detected using an antigen panel consisting of the *Pk* orthologues of MSP10, P12 and P38 (Muller-Sienerth et al., 2020).

Other malaria non-falciparum antigens, mainly from *Pv*, were also proposed as targets for serological surveillance due to their prevalence in malaria-endemic areas [reviewed in (Folegatti et al., 2017)]. Among them are *Pv* Apical membrane antigen-1 (PvAMA-1) with 63% (Sanchez-Arcila et al., 2015), PvMSP9 74% (Lima-Junior et al., 2008), PvMSP3 78% (Lima-Junior et al., 2011), *Pv* Duffy binding protein (PvDBP) 67% and *Pv* Reticulocyte binding protein-1 (PvRBP1) 66% (Tran et al., 2005) of prevalence in serum samples from individuals with confirmed *Pv* infections, respectively. Some of them were tested in ELISA assays, such as PvAMA-1 and PvDPB, but their performance for the species-specific detection or sensitivity was lower than PvMSP1_19_ (Barbedo et al., 2007).

Recently, Longley et al. (2020) reported a massive screening of *Pv* proteins in order to detect serological markers of recent infections potentially caused by relapses. This serological testing was performed using 342 samples from malaria-endemic regions in Thailand, Brazil and the Solomon Islands. The authors detected eight antibody responses which could serve as biomarkers, and suggested through mathematical models that treatment strategy with anti-hypnozoite therapy could reduce *Pv* prevalence by 59–69% (Longley et al., 2020).

Bead array-based technologies are also being explored as immunological malaria diagnostic methods. In one study, nine antigens from *Pf* and CSP from *Pm* were selected to obtain a multiplex Magnetic Bead-based MAGPIX^®^- Luminex Assay (MBA). In this study, only samples from *Pf*-infected patients were assayed, and the seroprevalence of antibody responses was highly variable (Koffi et al., 2015). In other approach, Mazhari et al. (2020) used a panel of 19 recombinant proteins from *Pv* coupled to non-magnetic and magnetic beads, in order to compare the performance of these methods when analyzed using two different instruments (Bio-Plex^®^ 200 and MAGPIX^®^). For the comparison, total IgG antibody levels against each recombinant protein were measured in plasma samples from 163 individuals living in malaria-endemic areas in Thailand and the Solomon Islands. The authors conclude that multiplexing assays performed using magnetic or non-magnetic beads are highly comparable, independent of the platform used to analyze the assays (Mazhari et al., 2020). Although these methods are relatively new and were not validated in field, these results are promising.

### Molecular Methods: Detection of iRBC

The most conventional method for malaria diagnosis, microscopy, uses visual detection of *Plasmodium*-infected RBCs, and as previously mentioned, this is still considered as the classical method of reference to date (World Health Organization, 2020). As an alternative to increase the accuracy of this type of approach, new methodologies have been gaining significance to fill the gap between visual and automatic detection of iRBC. Among them, flow cytometry-based approaches undoubtedly have potential as a malaria diagnostic method (Shapiro et al., 2013).

Most strategies focusing on the detection of *Plasmodium*-iRBC using flow cytometry are based on recognition of parasite forms (ring, trophozoite, and schizont) by analysis of scattergrams (Wongchotigul et al., 2004). This approach, improved with the use of fluorescence, demonstrated high sensitivity and was validated using blood samples from patients with *Pf* (n = 122) and *Po* (n = 2) malaria. Nevertheless, this method does not discriminate among the *Plasmodium* spp present in the sample; infections were categorized as *Pf* or “others”. This classification is based on RBC infected with *Pf* containing small ring forms compared to non-falciparum spp, and thus generates a distinct light scatter pattern (Pillay et al., 2019). Further validation is necessary to establish sensitivity to all non-falciparum spp.

*Plasmodium* spp-specific detection through flow cytometry requires the use of monoclonal antibodies recognizing some of the *Plasmodium* species-specific proteins present in the iRBCs. For the specific detection of *Pv*-infected RBCs, Roobsoong et al. (2014) developed an antibody-based flow cytometry assay. The combination of pan-malarial anti-PfBip antibody and *Pv*-specific anti-MSP1, anti-DBP and anti-Pvs16 antibodies conjugated to different fluorophores enabled the quantitative detection of *Pv*-infected RBCs (Roobsoong et al., 2014). As the assay was developed for laboratory research purposes, it was not field-validated.

Although flow cytometry as a diagnostic method has high sensitivity, its implementation in the field would require well-trained technicians. Moreover, the required equipment is expensive, which can represent a critical issue in low-resources settings. An alternative could be the use of portable, low-cost cytometers, which have been tested only for *Pf* detection (Yang et al., 2017), thus requiring adaptations for the application to non-falciparum spp.

Finally, a recently developed desktop instrument, the Parasight (Sight Diagnostics, USA), uses digital fluorescence microscopy and computer vision algorithms for malaria diagnosis. In brief, the instrument mimics the work of an expert microscopist, using vision algorithms created from control samples as “experience/training”. In this way, the program is able to detect parasitized RBCs, and differentiate among different stages and *Plasmodium* spp. The method proved to be highly sensitive, as it utilizes a combination of DNA and RNA fluorescent dyes. Nonetheless, some samples with levels of parasitemia below 20 parasites/μL were not detected, and several *Pf* cases were erroneously identified as *Pv*, both by the local microscopists and by the device. The errors were caused by large trophozoites that resembled *Pv* instead of *Pf*. In the same way, the device was unable to distinguish between *Pv* and *Po* (Eshel et al., 2017). This demonstrates that some human errors in species identification can be replicated by instruments following an algorithm. Nonetheless, Parasight was the better performing method compared to RDT and microscopy, being able to specifically detect all *Pf* (n = 5) and *Pv* (n = 19) infected samples, confirmed by PCR. The method also detected two mixed (*Pf* + *Pv*) infections (Das et al., 2020). In addition to the need of validation in the field or with higher number of samples, this methodology requires expensive equipment. As mentioned for cytometers, this issue can be critical for implementation in low-resource settings.

### Molecular Methods: Detection of Nucleic Acids by PCR

Molecular techniques have been widely used for the diagnosis of infectious diseases, including malaria. Molecular tests consisting of amplification and detection of specific sequences of DNA or RNA of the parasitic blood stages are recognized for their high sensitivity and specificity, PCR being the most used in diagnostic laboratories (Ayong et al., 2019).

As described in **Table 1**, despite all of the advantages related to PCR-based techniques, its use remains mostly restricted to the laboratory environment, due to the high costs of the reagents and equipment, the need for qualified professionals for execution and care to avoid contamination. Thus, the use of PCR-based techniques is often limited to reference laboratory settings and for research purposes (Ayong et al., 2019). The possible exception is the Loop-Mediated Isothermal Amplification (LAMP) technique, as it requires minimal equipment and reaction preparation, and its basic instructions and results are more easily interpretable (Becherer et al., 2020). For these reasons, LAMP has the potential to be used in field conditions (this method will be explored in detail in another section).

While the PCR method is not yet completely field-applicable, for the goal of malaria control and elimination this technique is of paramount importance to detect the right species and to give the proper treatment to asymptomatic individuals. PCR allows for sensitive and specific analysis being, until now, the only diagnosis method for malaria which can be routinely employed and capable of detecting asymptomatic submicroscopic infections. For instance, in the Peruvian Amazon, using qPCR, there was a prevalence of asymptomatic infections in 25% of the samples (585 for *Pv* and 122 for *Pf*/2,768 total samples); of them, 616 (87.1%) were light microscopy negative, i.e. submicroscopic parasitemia (Carrasco-Escobar et al., 2017).

Typically, targets in molecular diagnosis are genes with high copy numbers in the genome of the pathogen. Historically, the most used target for malaria detection by PCR is the coding region of the minor subunit of ribosomal RNA (18S rRNA), a target with 5-8 copies/parasite, though it was proposed that the use of multiple molecular targets could increase the chances of detecting submicroscopic malaria infections (Amaral et al., 2019). However, most sequences identified as promising molecular targets are specific to *Pf*. Therefore, for the specific detection of non-falciparum infections, 18S rRNA is still the most used target; either through the use of species-specific primers or by genus-specific PCR amplification followed by post-PCR methods to distinguish species (Zimmerman and Howes, 2015). This and other targets evaluated for molecular diagnosis of non-falciparum species will be addressed here.

Most strategies focusing on molecular methods to diagnose human malaria were developed or field-validated before the recognition of *Pk* as human-infecting species. Early studies showed high sensitivity of PCR and nested-PCR techniques using the 18S rRNA sequences to detect the different *Plasmodium* species in human samples, even detecting mixed infections that were missed by microscopy analyses (Snounou et al., 1993a; Snounou et al., 1993b; Singh et al., 1999). To date, nested-PCR is considered the gold-standard method for detecting sub-microscopic infections (see **Table 1**). Also, most comparative studies analyzing specificity and accuracy use this technique as reference for comparison. A disadvantage of this method is that it requires multiple PCR assays to be performed on each sample for species determination in mixed infections. Moreover, when the assay is performed using *outer* primers based on pan-*Plasmodium* sequences, there is a risk of disproportional amplification of the predominant specie (frequently *Pf*) and thus the less prevalent could be missed. In addition, this method does not provide quantification.

The sensitivity of Real Time-PCR (qPCR), which allows for parasite diagnosis and quantification, is similar to that obtained using nested-PCR, with a LOD of 0.7, 4, and 1.5 parasites/μL for *Pf*, *Pv* and *Po*, respectively (Perandin et al., 2004). Multiplex (duplex) qPCR assays using the 18S rRNA sequences proved to be a useful strategy in detecting *Pf, Pv* and *Pm* single or mixed infections, with equal sensitivity and specificity to nested-PCR, while simplifying the process and reducing costs (Veron et al., 2009). Moreover, this methodology was applied on re-evaluation of *Pf*- and *Pv*-infected samples diagnosed by microscopy using dried blood spots. The results confirmed that the diagnosis efficiency of microscopy is very low for species-specific and mixed infection detection (Tajebe et al., 2014).

The specific differentiation between *Po curtisi* and *Po wallikeri* required the use of optimized primer and probe sequences that improve sensitivity and specificity of molecular detection assays (Phuong et al., 2014). Similarly, a strategy to distinguish between *Po* and *Pm* uses minor groove binder (MGB) probes to overcome the shortness of the non-polymorphic region of 18S rRNA sequences (Rougemont et al., 2004). In other assays, MGB probes were used to estimate the parasite density in terms of parasite/μL. Thus, one copy of plasmid DNA was found to be equivalent to 0.281 and 0.127 parasites/μL parasites for *Pf* and *Pv*, respectively (Kamau et al., 2013). The multiplex assay described by Rougemont et al. (2004) for simultaneous identification of all four *Plasmodium* species (Rougemont et al., 2004) was successfully modified by Shokoples et al. (2009) in order to improve its ability to detect mixed infections (Shokoples et al., 2009). These primers were used for validation performing RT-qPCR on 77 clinical samples, obtaining 100% sensitivity to both *Pf* and *Pv* (Gavina et al., 2017).

A single qPCR-SYBR Green detection assay based on the amplification of *Pf*, *Pv*, *Po* and *Pm* species-specific regions of the 18S rRNA gene was developed, allowing detection of infected samples with 0.01 to 0.02% parasitemia. Analytical sensitivity was estimated to be 0.2 genome equivalent per reaction. The assay could specifically detect 97.4% of samples confirmed by microscopy, correctly identifying *Pv*-infected samples which were mistakenly detected as *Pm* and *Pf* infections (Mangold et al., 2005).

A limitation of the classical qPCR technique is the dependence on the use of fluorophores or DNA intercalating fluorescent dyes. To overcome this, self-quenching primers can be used in a procedure called photo-induced electron transfer (PET) PCR. This method was applied for the specific amplification of the *Plasmodium* genus and *Pf* species and validated using 119 clinical samples of different malaria species infections, including mixed infections. The limits of detection were 5.8 parasites/μL for *Po*, 3.5 parasites/μL for *Pm* and 5 parasites/μL for *Pv* using the genus-specific primer set (Lucchi et al., 2013). The sensitivity and specificity of this method were field-validated with samples from different regions, demonstrating similar performance to nested-PCR, while less expensive and easier to use (Talundzic et al., 2014; Escobar et al., 2020).

A multiplex PCR-ligase detection reaction based on the amplification of the gene-specific sequences of the 18S rRNAs, allowed the simultaneous diagnosis of infection by four parasite species (*Pf, Pv, Po, Pm*) with sensitivity of 1 parasitized erythrocyte/μL of blood (McNamara et al., 2004). This assay was further improved by the addition of probes that were bound covalently to fluorescent microspheres, enabling semiquantitative detection through dual fluorescence flow cytometry. The assay would be capable of providing a semiquantitative estimate of parasitemia between 0.3 and 1,000 iRBCs/μL (McNamara et al., 2006). A subsequent validation of this assay showed 85–93% concordance for *Plasmodium* spp. diagnosis compared with microfilarial positivity in blood samples (Mehlotra et al., 2010). Recently, this assay was used as a molecular diagnosis of *Plasmodium* species for analyzing the microscopy and RDTs performance: of 526 samples PCR-positive for *Pf*, 40 for *Pv*, 12 for *Pm*, and 8 samples for *Po* infections; 16.3% of *Pf*, 70% of *Pv*, 100% of *Pm*, and 100% of *Po* infections were submicroscopic. Also, 74.2% of non-falciparum infections and 37.5% of mixed-species infections were not detected by RDTs (Mehlotra et al., 2019).

Absolute quantification of parasites detected by qPCR is difficult to achieve, as it requires standard curves and DNA extraction that is rarely efficient, therefore it makes the comparison of qPCR results across laboratories less accurate. To overcome those difficulties, Koepfli et al. (2016) reported the use of droplet digital PCR (ddPCR) as a strategy that allows absolute quantification of 18S rRNA from *Pf* and *Pv*. The assay demonstrated higher sensitivity than qPCR to diagnose *Pf* and equal sensitivity for *Pv*, whereas precision in quantification was higher in both cases. Also, ddPCR was more sensitive to diagnose mixed infections (Koepfli et al., 2016). In another study, a sensitive assay using ddPCR was developed for detection and quantification of 18S rRNA of *Plasmodium* genus and species (*Pf, Pv, Po, Pm*). In artificially mixed samples with *Pm* as a minor population against a background of high parasitemia of *Pf* or *Pv*, ddPCR was able to detect *Pm* through duplex *Pf/Pv* and *Pm/Po* detection, whereas qPCR could not.

The diagnostic sensitivity and specificity of these ddPCR assays were validated using 32 DNA samples obtained from asymptomatic malaria patients, in which qPCR could not determine the specific species. The duplex ddPCR for specific *Plasmodium s*pecies detection was able to identify all 32 samples as 12 *Pv*, 16 *Pm*, 3 mixed *Pv* + *Pm* and 1 mixed *Pm* + *Po* infections (Srisutham et al., 2017).

Given that saliva and urine could be promising non-invasive samples, PCR assays were also performed to detect 18S rRNA sequences in those samples from symptomatic malaria patients. Even with 100% specificity, sensitivity was 74.1 and 84% (saliva), and 44.4 and 34.0% (urine), for *Pf* and *Pv* detection, respectively (Buppan et al., 2010).

In molecular diagnosis of *Pk* infections, amplification of the species-specific 18S rRNA was also described in early studies, both using PCR and qPCR (Cox-Singh et al., 1997; Singh et al., 2004; Babady et al., 2009). A qPCR assay using Pk8 and Pkr9 primers was clinically validated for the specific detection of *Pk*-infected individuals, demonstrating excellent sensitivity and linearity (Divis et al., 2010), furthermore, a single-step hexaplex PCR system targeting *Pf, Pv, Po curtisi, Po waliikeri, Pm* and *Pk* 18S rRNAs was developed. The assay, validated in 184 clinical samples, successfully detected all five human malaria parasites and mixed infections (Chew et al., 2012). However, a concern was raised when the Pk8 and Pkr9 primers were reported to cross-react with *Pv*, leading to potential false positive results (Imwong et al., 2009). Other *Pk*-specific primers based on the 18S rRNA were developed [reviewed in (Singh and Daneshvar, 2013)] as well as other multi-copy DNA sequences in *Pk* genome, such as Pkr140, were described as relevant targets for molecular diagnostic tests, demonstrating improved performance, such as the absence of cross-reactivity with other *Plasmodium* spp including *Pv* (Lucchi et al., 2012). Also, a semi-nested multiplex PCR method was able to detect *Pf, Pv, Pm* and *Po* infections (Rubio et al., 2002) and was further modified by adding *Pk*-specific sequences and validated against laboratory and clinical samples. The *Pk*-assay demonstrated 100% of specificity, as no cross-reactivity was found with any other human-infecting *Plasmodium* spp in 80 samples, including mixed infections (Van Hong et al., 2013).

Other diagnostic targets, potentially more sensitive than 18S rRNA, were also suggested for *Pf* (Pfr364) and *Pv* (Pvr47) detection (Demas et al., 2011), including a 28S rRNA (Pakalapati et al., 2013) and non-ribosomal multi-copy sequences useful to identify low-density, mostly asymptomatic *Pv* and *Pf* infections (Amaral et al., 2019). For the specific detection of *Po*, primers targeting the *Po*-RBP2 gene were proposed (Miller et al., 2015). In another study, a PET-PCR assay using those primers demonstrated a sensitivity and specificity of 97.5% and 99.2% for the detection of *Po* in 173 clinical samples, with a LOD of 1 parasite/μL (Akerele et al., 2017).

*Plasmodium* mitochondrial DNA (mtDNA) is another type of sequence used for malaria diagnosis through PCR assays, due to the high copy number/cell of the parasite mitochondrial genome, from 20 to 150 copies/parasite. For this reason, mtDNA as target has the potential to be more sensitive than other genomic sequences. In the first report suggesting this strategy, specific primers were designed to hybridize with mitochondrial cytochrome c oxidase III (Cox3) genes of *Pf* and *Pv*. The applicability of the PCR assay was analyzed in a cross-sectional study, using 88 randomly selected samples from individuals naturally exposed to malaria. Sensitivity and specificity were 100% and 88.3%, respectively, and the assay was able to detect submicroscopic *Pv*-infections, showing that 23% of the randomly selected samples of healthy individuals from an endemic area were asymptomatic carriers (Cunha et al., 2009). Considering the serious implications for the potentially undetected malaria transmission in endemic areas, an improved method (Cox3 amplification by qPCR with TaqMan probes) to detect *Pv*-infections was applied in samples from 595 blood donors collected in areas at risk for *Pv* malaria transmission. The assay was able to identify *Pv* in 1.34% of clinically healthy donors, highlighting the potential risk for transfusion-transmitted malaria (Batista-dos-Santos et al., 2012). This methodology was further developed for enabling the detection of *Pf, Pv* and *Pm* in blood samples, and proposed as a strategy to improve malaria surveillance preventing transfusion−transmitted malaria in blood banks (Batista-Dos-Santos et al., 2018).

Echeverry et al. (2016) developed a direct single PCR assay for the detection of Cox3 pan-*Plasmodium* sequences. The performance of this assay was compared to 18S rRNA direct nested-PCR as a reference, obtaining better results regarding LOD (0.6–2 parasites/μL for Cox3 vs. 2–10 parasites/μL for 18S sequences). However, the Cox3 direct PCR was prone to detect only the predominant species in mixed infections (Echeverry et al., 2016).

In another approach, Isozumi et al. (2015) developed a Cox3-based nested-PCR requiring only a single round of amplification for specific detection of *Pf*, *Pv*, *Po* and *Pm* DNA in blood samples collected from field. The Cox3-PCR showed higher overall efficiency compared to other mtDNA and to standard 18S rDNA-PCR, significant for the detection of microscopically negative samples and mixed infections (Isozumi et al., 2015). Based on these results, a multiplex single-tube nested-PCR targeting also *Pk* was developed and combined to a single-stranded tag hybridization (STH) chromatographic printed-array strip (PAS) method. The assay was validated with samples from malaria-infected individuals and the assay’s sensitivity and specificity were 88.7% and 100%, respectively (Saito et al., 2018). Although this method requires further validation, it has the potential for developing a PoC test.

Other mtDNA, cytochrome b (CytB) gene, was proposed as a target for *Pf, Pv*, *Po* and *Pm* detection in a comparative study where it proved to be more sensitive than 18S rRNA (Steenkeste et al., 2009). In agreement, a qPCR assay targeting the CytB gene of *Pf*, *Pv*, *Po* and *Pm* followed by *Plasmodium* species determination using RFLP analysis, performed equal to, or better than the reference 18S-PCRs with a sensitivity of 100% (65/65) and a specificity of 99.9% (2910/2912). The LOD of the CytB-qPCR was 1 parasite/μL for *Pf* and *Po*, and 2 parasites/μL for *Pv* and *Pm* (Xu et al., 2015).

A nested-PCR method detecting CytB of the five *Plasmodium* species, including *Pk*, demonstrated 16% and 39.8% more sensitivity than 18S-PCR and microscopy, respectively, in detecting all these malarial species in blood samples. Moreover, this assay was able to detect 34% and 17% of mixed *Pf* and non-falciparum infections, initially classified by microscopy as *Pf* and *Pv* monoinfections, respectively. Though less sensitive than blood-based molecular assays, CytB-PCR performed with saliva samples was more sensitive than microscopy for diagnosis of mixed-species infections, identifying *Pm* and *Pk* species when other techniques could not (Putaporntip et al., 2011).

A single-round PCR assay was also developed using mitochondrial targets to detect *Plasmodium* spp, with a sensitivity of 97% at the threshold dilution 0.5 parasites/μL; a better performance than conventional PCR using 18S rRNA as target (Haanshuus et al., 2013). This assay was field-validated, demonstrating its usefulness in detecting submicroscopic infections, which elevated the malaria prevalence from 6% to 19% in hospitalized patients with acute undifferentiated fever (Haanshuus et al., 2016). In further works, the conversion from conventional CytB-based PCR to qPCR was assayed using either SYBR Green or TaqMan probe and comparing the performances to seven other qPCR methods. Both protocols showed high sensitivity and specificity, though the SYBR protocol was better-performing (Haanshuus et al., 2019).

TaqMan probes were also used in a recent study developing primers for qPCR to amplify *Pm/P. brasilianum*-specific CytB, achieving a LOD of 0.5-1 parasite equivalent/μL and 100% sensitivity and specificity among known *Pm*-infected samples (Dos Santos et al., 2020). CytB was also proposed as a marker for molecular discrimination between *Po curtisi* and *Po wallikeri*, among other markers including 18S rRNA and PoMSP-1 (Fuehrer and Noedl, 2014). Results using quantitative, nested, single-step PCR and PCR-RFLP methods validated that CytB was the better performing target (Zaw and Lin, 2017).

There are also other targets for molecular detection of *Plasmodium* spp described; some of them were included in multiplex assays. An example is the combined detection by qPCR of 18S rRNA for *Pf*, PvAMA-1 for *Pv*, and species-specific plasmepsin for *Po*, *Pm* and *Pk*. This assay showed clinical sensitivities of 95.8%, 89.5%, 94.1% for *Pf, Pv* and *Po* detection, respectively, and 100% for *Pm* and *Pk*. The specificities ranged from 98.4% to 100%, detecting from 1 to 6 parasites/μL of blood (Reller et al., 2013). The same markers were also used for the duplex detection of *Pv* (AMA-1) and *Pk* (plasmepsin) by ddPCR. The assay demonstrated high analytical sensitivity (*Pv* = 10 copies/μL and *Pk* = 0.01 copies/μL); however, clinical sensitivity and specificity were lower than those of qPCR (Mahendran et al., 2020).

Other multiplex qPCR assay suitable for detection of the five *Plasmodium* spp combined different targets: the previously described Pfr364 for *Pf* (Demas et al., 2011) and Pkr140 for *Pk* (Lucchi et al., 2012), and newly designed primers for the specific amplification of dihydrofolate reductase (dhfr) genes for *Pv*, *Pm* and *Po* (Lefterova et al., 2015). Clinical sensitivities calculated using 52 samples were 95.2% for *Pf* and 100% for all non-falciparum spp. This assay was included as a PCR method for comparison in a previously described study (Haanshuus et al., 2019) and further used for confirmation of malaria species identification in samples from febrile patients (Leski et al., 2020).

Usually, in-house qPCR approaches are associated with lower costs than commercial test kits, however, for accredited diagnostic laboratories, commercial kits are more appropriate, since their procedures are standardized and contain quality-controlled components (Frickmann et al., 2019). The first commercially available qPCR assay for the diagnosis of malaria (RealArt Malaria LC PCR assay) proved to be rapid, sensitive and clinically specific for the detection of malaria parasites in febrile returned travelers. Nevertheless, the commercial kit was unable to differentiate among *Plasmodium* spp (Farcas et al., 2004).

Other commercial kits developed in recent years, were able to detect and discriminate among species. One of them, (PlasmoNex™), based on the hexaplex qPCR assay previously described (Chew et al., 2012), was compared to species-specific qPCR and proved to be the most specific and sensitive detection technique (100%) when confronted with sequencing results. The kit was able to detect mixed infections of *Pv*, *Pf*, and *Pk*, which were further confirmed *via* sequencing. Neither *Pm* nor *Po* were detected among the samples (Lee et al., 2015).

The performance of two other commercial kits (RealStar Malaria S&T PCR Kit 1.0 and FTD Malaria Differentiation) was analyzed and compared to species-specific qPCR, using 247 blood samples positive for *Plasmodium* spp (Frickmann et al., 2019). Results showed a concordance of 98.9% in the species identified, compared to microscopy, and 95.1% (RealStar) and 96.8% (FTD) compared to genus-specific PCR. Comparatively, RealStar kit revealed slightly reduced sensitivity for submicroscopic *Pf* infections, whereas it was better-performing than FDT in the detection of *Pk* and non-falciparum spp with low-level parasitemia. Both commercial kits were able to detect mixed and *Po* infections misidentified as *Pv* in microscopy. The excellent performance of these kits was further confirmed in a meta-analysis when comparing both kits with a species-specific in-house qPCR and a LAMP method using the Meridian *illumigene/Alethia* malaria platform (Altangerel and Frickmann, 2020) which will be described in the next sections.

## Potentially New Point-Of-Care (POC) Methods

In a recent review, Rei Yan et al. (2020) summarized the requirements for PoC tests for malaria diagnosis, considering that most of the malaria-vulnerable population from endemic areas lives in remote regions where medical assistance and resources are scarce. For these reasons, an optimal PoC test for malaria diagnosis must be cheap, suitably performed near the patient, does not require specialized equipment or complicated technical training, and provides good sensitivity. Although neither biosensors, nor molecular approaches, would fulfill all of those requirements, there are techniques with the potential to be implemented in low-resource laboratories and can be considered as PoC approaches (Rei Yan et al., 2020). Among them are aptamer-based assays, electrochemical or microfluidic devices, and other ultrasensitive devices that will be discussed in the following section, whereas portable PCR assays and LAMP-based techniques for the specific detection of non-falciparum malaria will be covered in the later section (see **Table 1**).

### Biosensors and Ultrasensitive Devices

Advances in nanotechnology and sensor systems in the last few years have prompted the development of novel diagnostic methods and detection systems with the aim of overcoming the obstacles of conventional diagnostic methods, regarding biosensors (Ragavan et al., 2018). Accordingly, immunosensors have been extensively studied in clinical diagnoses where biological sensing is integrated with microfluidic devices in an attempt to obtain promising sensing tools with several analytical benefits for malaria detection (Ruiz-Vega et al., 2020; Dip Gandarilla et al., 2021). Among these types of sensors, electrochemical and optical biosensors, with labeled or label-free detection, have received considerable interest in clinical diagnostics due to their assay simplicity, and superior analytical performance over conventional laboratory methods, showing low detection limits, wide linear range, stability and reproducibility.

As mentioned previously, high sensitivity is especially necessary for non-falciparum malaria detection, as low parasitemia is frequent among patients (Antonelli et al., 2020). Most of the sensors described to date for malaria diagnosis only detect *Pf* and occasionally *Pv*, using the same antigens as RDTs (Krampa et al., 2020). Thus, the detection of *Plasmodium* non-falciparum, and particularly non-falciparum non-vivax species, is largely neglected.

Several authors developed devices to detect *Pf* and *Pv* simultaneously. Lee et al., 2012 described an electrochemical aptasensor development for malaria, based on pLDH protein detection. The system is composed of an aptamer bound by thiol groups on a gold electrode, the signal output being carried out on a platform of impedance electrochemical spectroscopy (EIS). The LOD reported for these aptasensors was 108.5 fM and 120.1 fM for PvLDH and PfLDH, respectively (Lee et al., 2012). Similarly, a colorimetric aptasensor based on the interaction of pLDH and pL1 aptamer against PvLDH and PfLDH was developed. The cationic polymers, poly(diallyldimethylammonium chloride) (PDDA) and poly(allylamine hydrochloride) (PAH) aggregate to gold nanoparticles (AuNPs), producing a color change from red to blue, depending on the pLDH concentration. The LOD for PvLDH was determined as 8.3 pM for PAH and 8.7 pM for PDDA. The LOD for PfLDH was 12.5 pM for PAH and 10.3 pM for PDDA. In addition, pLDH from infected blood samples were detected for *Pv* as 74 parasites/μL for PAH and 80 parasites/μL for PDDA, and for *Pf* as 97 parasites/μL for PAH and 92 parasites/μL for PDDA (Jeon et al., 2013).

Another colorimetric aptamer diagnostic test to discriminate *Pf* from *Pv* was also reported by Cheung et al. (2018). The test uses two different aptamers which binds to PfLDH, although through different mechanisms. While the pL1 aptamer bound with high affinity to both PfLDH and PvLDH, the 2008s aptamer binds specifically to PfLDH. Thus, a sensitive aptamer-tethered enzyme capture (APTEC) assay specific for *Pf* was developed. In malaria patient blood samples, the 2008s APTEC assay was specific for *Pf* detection and could discriminate *Pv* (Cheung et al., 2018). On the other hand, an impedimetric biosensor to specifically detect PvLDH was developed, based on the anti-pLDH antibodies chemically cross-linked *via* glutaraldehyde to interdigitated electrodes (IDEs). PvLDH was detected by recording the sensor of the electrical signal before and after the sample addition. The sensor detected recombinant PvLDH (spiked in PBS) at concentrations as low as 250 pg/mL (Low et al., 2019).

In order to obtain a *Pf*/*Pv* differential diagnosis, Ittarat et al. (2013) used a quartz crystal microbalance (QCM). The QCM silver electrode surface was sequentially modified with avidin and malaria biotinylated probes, which binds specifically with amplified malaria-infected blood DNA fragments, resulting in quartz frequency shifts. The QCM device was able to differentiate blood infected with *Pf* from that infected with *Pv*, without cross-reaction with human DNA. This method was evaluated with blood samples from malaria-infected individuals previously diagnosed by microscopy and RDTs. The device was able to detect 30/30 samples with consistent results compared to RDTs performance; while microscopical examination detected 27/30 *Pf* infections (Ittarat et al., 2013). In the same way, Wangmaung et al. (2014) developed a QCM to identify both single and mixed *Pf* and *Pv* infections. The biotinylated malaria probe was immobilized on a silver QCM surface *via* specific avidin-biotin interaction. DNA target fragment of 18S rRNA genes species-specific sequences were amplified and hybridized with the immobilized malaria probe. The detection is based on mass changes due to hybridization, which shows changes in quartz resonance frequencies. Out of a total of 67 blood samples from malaria-endemic areas, this device was able to specifically detect *Pv* (n = 26), *Pf* (n = 25) and mixed infections (n = 2). The results were consistent and demonstrated that QCM identifies false-negative and misdiagnosis routine cases (Wangmaung et al., 2014).

Regarding the specific *Pv* detection, Cardoso et al. (2017) reported a biosensor that enables PoC detection of antibodies against two *Pv* antigens, Circumsporozoite protein (CSP) and Thrombospondin related anonymous protein (TRAP). The device consists of a carbon working electrode modified with carbon nanotubes (CNTs), followed by an EDC/NHS treatment and the *Pv* protein fragments immobilization. The antibodies present in serum samples were detected by electrochemical impedance spectroscopy (EIS). Linear ranges were observed at antibody concentrations from 50 pg/L to 70 μg/L (Cardoso et al., 2017). Likewise, recently Regiart et al. (2021) developed a microfluidic electrochemical immunosensor (Regiart et al., 2021) for detection of antibodies against PvMSP1_19_, using for comparison an ELISA assay previously described (Rodrigues et al., 2003). This assay enables detection of *Pv* new infections and relapses as well. The biosensor consist of a gold microelectrode used as immobilization platform, modified by the dynamic hydrogen bubble template (DHBT) method in the presence of multiwalled carbon nanotubes. The detection limit of this microfluidic electrochemical device was 0.6 ng mL^-1^ of the antibody (Regiart et al., 2021). Focusing on the specific detection of *Pv*-infected patients, Singh et al. (2021) also recently developed a PoC device for electrochemical detection of MSP1 in *Pv*-iRBCs. The device is based on the synthesis of reduced graphene oxide-gold nanocomposite (Au-rGO), followed by MSP1 antibody immobilization with ethylenediamine (EDA), and deposition over a carbon strip. The sensor showed an increase in charge-transfer resistance in ferro/ferricyanide solution due to the interaction between MSP1 antibody and *Pv*-iRBCs, showing a LOD of 4 iRBCs/μL blood sample (Singh et al., 2021).

To our knowledge, the only biosensing device developed for specific detection of *Pk*-infections was reported by Shah et al. (2017). The strategy consists of using fluorescence *in situ* hybridization (FISH) assay to detect positive cases, using a *Pk*-specific 18S rRNA sequence-based DNA probe. The results showed that this device could easily discriminate *Pk* in mixed infections with *Pf* and *Pm*. Although the technique is simpler than PCR-based tests, it requires filter attachments and a fluorescence microscope, which is a limiting technical aspect for PoC devices. The LOD for *Pk* was 84 parasites/μL in blood samples from infected monkeys and 61 parasites/μL for cultured human blood samples, comparable to microscopy technique (Shah et al., 2017).

Finally, a CRISPR (clustered regularly interspaced short palindromic repeats)-based method for differentiation and detection of *Pf*, *Pv*, *Po*, and *Pm*, was recently developed (Lee et al., 2020). The new method is based on the nucleic acid detection SHERLOCK (specific high-sensitivity enzymatic reporter unlocking) platform. The system includes a rapid (10 minutes) extraction of parasite DNA, followed by *Plasmodium* species-specific detection *via* fluorescent or lateral flow strip readout. The LOD was 2 parasites/μL. The *Pf*- and *Pv-*specific assays exhibited 100% specificity and sensitivity on clinical samples, demonstrating promising results.

Despite their promising results, to our knowledge, all of these biosensing devices and new methodologies remain to be validated in the field.

### Field-Applicable Molecular Approaches

As previously discussed, the huge utility of PCR-based approaches in detecting submicroscopic parasitemia, and to accurately identify non-falciparum or mixed infections, has led to efforts in adapting this technique into portable, faster, and easier-applicable tests. In regards to portability, a mobile laboratory was implemented for performing DNA extraction and qPCR assays, in order to detect asymptomatic infections in a low transmission area. The proposed procedure, a two-step PCR-qPCR for detection of *Plasmodium* (genus) infections and determining species (*Pf, Pv, Po*, and *Pm*) respectively, allowed the detection of 4.9% of the population evaluated (244/4,999 samples) as asymptomatic carriers of *Pv* (49.2%), *Pf* (34.0%), *Pm* (3.3%) and *Po* (0.4%) single infections, and 13.1% of mixed infections, using CytB (genus and spp-specific) amplifications.

The main limitations of this approach were related to the blood-volume able to be analyzed, and the correlated DNA extraction step that restricted the LOD to 2 parasites/μL (Canier et al., 2013). This approach also, excluding the portability of the laboratory, presents other limitations common for PCR methods previously explained including the necessity of cold-chain for a stable and controlled temperature of -20°C during transport and storage of reagents.

In order to overcome the cold-chain requirement, a previously mentioned multiplex malaria qPCR assay (Kamau et al., 2013) was lyophilized by freezing and drying all qPCR components, creating the Malaria Multiplex Sample-Ready™ (MMSR) format. This assay, stable at room temperature (RT) and at 37°C for at least 42 days, requires only the addition of water and a sample to perform qPCR (Kamau et al., 2014). The performance of the MMSR assay was further evaluated to test blood samples from febrile patients in Sierra Leone (Leski et al., 2020), being able to identify *Pf* and *Pv* at species level. The assay also detected other samples as non-falciparum non-vivax infections, which were identified by another multiplex assay (Lefterova et al., 2015) as *Po* and *Pm* infections.

An automatic portable micro-qPCR analyzer, Truelab^®^ Uno, was adapted to specifically detect malaria through the utilization of Truenat^®^ microchips, containing pre-loaded, RT-stabilized PCR reagents. The performance was assayed using *Pf*- and *Pv*-infected samples, demonstrating a LOD of 4.7 parasites/μL for *Pf* and an estimated 1.3 parasites/μL for *Pv*. The total time assay was ~1 hour/sample and the sensitivity at the species level was 99.3%, compared to microscopy, with the advantage of detecting mixed and submicroscopic infections (Nair et al., 2016).

A lab-on-chip qPCR diagnostic platform for malaria named Accutas system was developed seeking portability at room temperature (Taylor et al., 2014). The platform consists of portable qPCR equipment and a disposable plastic chip containing all reagents needed for *Plasmodium*-specific qPCR desiccated in hydrogel. The assay can be performed directly with unprocessed blood, avoiding the need for sample preparation, and has a high sensitivity (97.4%) for the detection of all *Plasmodium* spp, though only differentiating *Pf* and *Pv*. Likewise, Rampazzo et al. (2019) developed a prototype platform comprising a portable device and a silicon chip named Q3-Plus for the detection of *Plasmodium* spp. and *Trypanosoma cruzi* infections through ready-to-use qPCR. The Q3-Plus system, able to detect *Plasmodium* at genus level and differentiate among *Pf*, *Pv* and *Pm* through species-specific hydrolysis probes, revealed the same sensitivity as assays performed with standard equipment (Rampazzo et al., 2019).

In summary, several “lab-on-a-chip” strategies for malaria diagnosis have the potential to fulfill the need for portable diagnostics with improved sensitivity. Nonetheless, they are focused on specific *Pf* detection and sometimes also *Pv* detection, but commonly neglect the specific detection of other *Plasmodium* spp (Kolluri et al., 2017).

As mentioned before, a molecular method with wide potential to be applied as PoC diagnostic for malaria is the LAMP assay. This method consists of isothermal amplification of the target DNA, without the temperature variation necessary for conventional PCR methods. This is achieved using a Bst polymerase, capable of synthesizing a new strand of DNA while dissociating the hydrogen bond from the template (strand-displacement activity). Moreover, one of the main characteristics of this technique is its high sensitivity and specificity, given by the utilization of 4-6 specific primers: two outer- (F3 and B3), two inner- (FIP and BIP) and two loop- (optional, LF and LB) primers. In summary, the assay can be performed using a temperature block instead of a thermocycler, is faster (~1h), and the visualization of results is also simpler since the detection of LAMP can be performed through measurement of the turbidity caused by precipitated magnesium pyrophosphate, endpoint detection with the naked eye, or a variety of quantitative methods (Becherer et al., 2020).

The specific detection of *Pf*, *Pv*, *Pm* and *Po* through the LAMP method was described for the first time by Han et al. (2007), with sensitivity of 98.5% and specificity of 96.7% compared to standard nested PCR. Positive samples were determined by observation of turbidity with the naked eye and confirmed with a Loopamp real-time turbidimeter (RT-160C; Eiken Chemical Co). LODs of 100 copies of the target 18S rRNA genes for *Pf* and *Pv* and 10 copies for *Pm* and *Po* were reported (Han et al., 2007). This method was field-validated, demonstrating sensitivity and specificity of 98.3% and 100%, respectively, compared to microscopy analysis (Sirichaisinthop et al., 2011). A research conducted in Thailand compared LAMP, microscopy and nested PCR for malaria diagnosis in blood samples. LAMP was able to detect 22/23 infections positive by nested PCR (reference technique) obtaining 100% sensitivity in infections caused by *Pv*, unlike microscopy that only detected 65% (Poschl et al., 2010).

In another study testing more malaria cases (*Pf* = 133; *Pv* = 124), LAMP was 100% sensitive and specific for *Pf*, whereas it exhibited 95.16% sensitivity and 96.7% specificity for *Pv* (Singh et al., 2017). LAMP assays for the specific detection of *Pv* only, targeting 18S rRNA, were also developed and evaluated using 145 microscopically confirmed *Pv* samples and 20 *Pv* negative patients. Sensitivity was 100% (LOD = 0.8 copies/μL) and specificity 85%, compared to microscopy (Kaur et al., 2018).

Species-specific LAMP methods based on 18S rRNA genes, specific for the detection of the five human-infecting *Plasmodium* (including *Pk*) were also developed. The sensitivity and specificity of a species-specific LAMP assay compared with microscopic examination and nested PCR were 100%, with a LOD of one copy for *Pf, Pv* and *Pm*, and 10 copies for *Po* and *Pk* (Lau et al., 2016). This assay was recently improved by the addition of the WarmStart colorimetric reagent, allowing the visual detection of LAMP products by direct observation of color changes. Clinical sensitivity (98%) and specificity (100%) were determined using 100 microscopy-positive and 20 malaria-negative samples. The test was even able to correctly detect *Pf*-infections in samples microscopically misdiagnosed as *Pm*-(7) and *Po*-(1) infections (Lai et al., 2020).

The main disadvantage of these strategies, however, is that a new LAMP assay must be performed on each sample in order to determine the presence of the specific *Plasmodium* spp. For this reason, commercial kits detecting *Plasmodium* 18S rRNA at genus level, and only *Pf* at species level, were developed.

The first commercial kit available in the market was LoopAmp Malaria (Pan/Pf) detection kit (Eiken Chemical Co). The kit was field-validated in samples from symptomatic patients, obtaining sensitivities far higher than microscopy but lower specificity, using PCR as a reference standard  (Hopkins et al., 2013; Polley et al., 2013). The performance was even excellent for detecting samples with confirmed non-falciparum malaria, such as 29 *Po*-positive (Cuadros et al., 2017) and 50 *Pk*-positive samples (Piera et al., 2017), with sensitivity of 100% and specificity of 97.24 to 100%, respectively. The LODs of the LAMP kit were between 0.8 and 2 parasites/μL for *Po, Pv* and *Pk* species. In order to analyze the actual usefulness of the kit as a malaria diagnostic tool in the field, an investigation was carried out in the Peruvian Amazon in 2017, comparing its performance to microscopy and reference qPCR.

The commercial LoopAmp Malaria (Pan/Pf) detection kit presented a sensitivity of 91.8% for any *Plasmodium* spp, high when compared to the microscopy with 20.3% sensitivity in the field (Serra-Casas et al., 2017). Nonetheless, the kit demonstrated a specificity of 91.9% compared to qPCR, evidencing the occurrence of false positives. In addition to the incapacity of differentiating among non-falciparum spp, samples detected as *Pf*- or *Pf*/mixed infections by qPCR were identified as non-falciparum by the commercial LAMP, or even not detected in low parasite density infections (below 4 parasites/μL). Despite these difficulties, the study suggested that a LAMP method would be a feasible surveillance tool for malaria screening campaigns in the Peruvian Amazon, since it was demonstrated that the training of unexperienced personal, spaces and time assay necessary to perform wide-scale LAMP testing was comparable to that of field microscopy while presenting higher accuracy and involving a much easier interpreted result (Serra-Casas et al., 2017).

With the purpose of facilitating malaria detection in low-resource settings, a non-instrumented nucleic acid amplification (NINA) heater developed by PATH (Seattle, USA), was used to implement the LoopAmp Malaria (Pan/Pf) detection kit for the diagnosis of malaria in Northwest Ethiopia (Sema et al., 2015). The results showed 96.8% sensitivity towards *Plasmodium* genus, however, the kit failed to detect 6/18 non-falciparum (*Pv* and *Po*) infections. An 84.3% specificity for detection of *Plasmodium* genus using nested PCR as reference was found, which indicates a ~15% rate of false positives, in line with previous studies. The cause is not clear, but contamination is very likely, since an improved version of a NINA coupled with the LoopAmp Malaria kit reported by the same group, showed better results (100% sensitivity and 98.6% specificity) for detection of traveler’s malaria (n = 140). The LODs were 5 parasites/μL for both Pan-*Plasmodium* and *Pf* primer sets (Mohon et al., 2016).

The other commercial kit available for LAMP-based malaria detection is the Meridian *illumigene/alethia* ^®^ Malaria platform (Meridian Bioscience Inc.). This kit uses LAMP-primers targeting a *Plasmodium* spp. Mitochondrial DNA noncoding region conserved across *Pf*, *Pv*, *Pm*, *Po* and *Pk*; therefore, the assay does not differentiate among *Plasmodium* species. In a retrospective analysis of stored blood samples from returned travelers, this kit presented sensitivity and specificity of 100% (n = 74 positives, 34 negatives), and analytical sensitivity equal to 0.5 parasites/μL for *Pf* and *Pv* samples (De Koninck et al., 2017). Similar results were obtained conducting the *illumigene/alethia* malaria test right after microscopic examination of 310 fresh blood samples from patients suspected of imported malaria. While sensitivity was 100% (*Pf*: 66, *Po*:9, *Pm*: 3, *Pv*: 3, *Pf + Pm*: 2), specificity was 93.64%, due to 4 false positives detected (Ponce et al., 2017).

In another study, the commercial kit was evaluated for routine malaria detection in blood samples of German travelers, with excellent results. Sensitivity was 98.7% (235/238); undetected samples were from two *Po*- and one *Pv*- infections. The LAMP kit provided false-positive results in 3/762 samples (0.4%), thus the specificity was 99.6% (Frickmann et al., 2018). The good performance of the *illumigene/alethia* malaria kit was also reported in a meta-analysis that assessed the diagnostic performance of this method compared to an in-house and the two commercial PCR kits previously mentioned (Altangerel and Frickmann, 2020). All four assessed molecular approaches led to concordant results in 988/1020 samples (96.9%).

Despite those results, limitations of these available commercial LAMP kits, such as the lack of species-specificity and quantification, promote the development and testing of in-house methods. For example, an approach using species-specific LAMP-primers seeking the specific determination of all five human-infecting *Plasmodium* was developed. Designed LAMP primers target the histone deacetylase gene sal-1 for *Pf*- and *Pk*-detection, dhfr gene for *Pv*, and previously published LAMP primers for the 18S rRNA gene for *Pm*- and *Po*-detection.

While the genus-specific in-house malaria LAMP identified 234/243 samples (sensitivity of 96.3%) in a performance comparable to the commercial *illumigene/alethia* malaria kit, the poor performance for the species-specific detection of *Pf* and *Pv* (sensitivities of 71% and 82.4%, respectively) led to the conclusion that the in-house malaria LAMP method lacks the reliability required for diagnostic laboratories (Kollenda et al., 2018). On the other hand, a successful and elegant strategy involved the amplification of the 18S rRNA gene of all five human *Plasmodium* species, including two *Po* subspecies, and then the identification of each *Plasmodium* species through the sequencing of the LAMP products using a MinION™ nanopore sequencer (Imai et al., 2017). The assay proved to be sensitive and specific for *Pf* (n=36) and *Pv* (n=17) detection, while the other species were not assayed. The strategy of sequencing amplicons for determination of the species present in malaria-positive human blood samples was also proposed using the Illumina MiSeq platform to quantify *Pf* and *Pv* by PCR (Wahab et al., 2020). However, this platform (mainly the required equipment) is relatively expensive and lacks desired characteristics for PoC settings. Differently, the MinION™ nanopore is a portable sequencer, easy to use, fast and a suitable complement for LAMP technique among others. In summary, it possesses wide potential to be a useful tool in the fight against malaria and other infectious diseases (Mongan et al., 2020).

Regarding quantification of positive samples, Lucchi et al. (2010) reported the generation of a portable device containing the heating block and the fluorescent (SYBR green) detection unit combined into a single, real-time assay, called RealAmp. This method was able to detect all five human-infective *Plasmodium* (*Pf, Pv, Po, Pm* and *Pk*) at genus level, using primers targeting *Plasmodium* spp 18S rRNA, with sensitivity of 96.7% and specificity of 91.7% compared to microscopy, and LODs of 10 parasites/μL (Lucchi et al., 2010). The RealAmp method was also used for the specific detection of *Pv* (Patel et al., 2013), through the generation of LAMP-primers targeting previously identified *Pv*-sequences Pvr47 and Pvr64 (Demas et al., 2011). These primers detected *Pv* in clinical samples with 94.6% of sensitivity and 100% specificity compared to standard nested-PCR (Patel et al., 2013).

Both RealAmp assays; i.e. the *Plasmodium* genus and *Pv*-specific detection, were evaluated as diagnostic tools for malaria in a posterior field study. Sensitivity ranged from 94.8 to 100%, and specificity from 96.7 to 100%, and *Pv*-specific LAMP was able to accurately detect all *Pv* cases. These results, and the short time of assay (30-75 minutes), suggested the potential of RealAmp to serve as a PoC test (Patel et al., 2014). In a posterior field evaluation of three independent RealAmp assays (*Plasmodium*-genus, *Pf* only, and *Pv* only), sensitivity (94.1%) and specificity (83.9%) were lower than those of the microscopy study when compared to reference-PCR assays. False positives were also reported for *Pf* (7/55 samples) and *Pv* (29/181 samples) and RealAmp method was also not able to detect any of the 20 mixed *Pf* + *Pv* infections (Viana et al., 2018). Nevertheless, a recent comparative study further assessed the performance of these three RealAmp methods and a field-validated malachite-green (MG)-LAMP methodology (Kudyba et al., 2019), using microscopy and nested-PCR as reference techniques. In this study, the two LAMP approaches were equally specific (100%) and more sensitive (92-94%) than microscopy (sensitivity of 88.1%) (Barazorda et al., 2020). Those apparent inconsistencies in the field-evaluation of LAMP methods could be due to the high probability of cross-contamination associated with this technique.

Similar to PCR-based assays, other diagnostic targets for the detection of non-falciparum malaria were also tested by LAMP. An example is species-specific α- and β- tubulin genes, assayed for the specific diagnosis of *Pv* and *Pk*, respectively. Sensitivity, specificity, and clinical applicability of *Pk*-specific β- tubulin as a target for LAMP, were 100% in the blood samples obtained from experimentally *Pk*-infected monkeys (Iseki et al., 2010). Regarding *Pv*, a LAMP assay targeting a *Pv*-specific sequence of the α-tubulin gene was developed and its diagnostic performance was compared to microscopy, RDTs and nested PCR in 177 samples. This assay was highly specific (100%) and more sensitive than either microscopy or RDTs. However, sensitivity compared to the gold standard nested PCR was 72.3% (Dinzouna-Boutamba et al., 2014).

Following the good PCR performance results on *Plasmodium* mtDNA sequences, these molecular targets were also assayed by LAMP methods. A pan-genus (PgMt19) and a *Pf*-specific (PfMt869) LAMP primer sets were designed for the detection of Pan/*Pf* malaria. The pan-genus assay amplified samples containing 5 parasites/μL from all five species, with sensitivity of 93.9% and specificity of 100% compared to nested PCR (Polley et al., 2010). In other approaches, the direct blood dry LAMP system (CZC-LAMP) was applied to malaria diagnosis, using two primer sets: one for mtDNAs *Pf*-specific and another for mtDNA non-falciparum (*Pv*, *Pm* and *Po*)-specific. The dry LAMPs performed better with anticoagulated blood as the template (sensitivities 98.1% for *Pf*, 92.1% for non-*Pf*) than purified blood DNA samples (Hayashida et al., 2017).

*Pv* mtDNA was also assessed as the target region for *Pv*-specific detection. In one assay, the LAMP method was modified to allow visualizing the results in the closed-tube with the use of microcrystalline wax-dye capsules. Compared to microscopy, the sensitivity and specificity of this *Pv*-specific LAMP assay were 98.3% (59/60) and 100% (29/29), respectively (Tao et al., 2011). In another study, a high throughput LAMP-*Pv* assay (HtLAMP-Pv) targeting *Pv*-COX2 gene was developed. Field testing was conducted using 149 samples from symptomatic malaria patients, and results showed a sensitivity for *Pv* of 95% (LOD = 1.4 parasites/μL). Specificity concerning non-detection of *Pf* and *Pm* samples was 100%, however, the primers presented cross-reaction with *Pk-*COX2, probably due to the high similarity between *Pv-* and *Pk*-COX2 sequences (Britton et al., 2016). The same group adapted the HtLAMP platform, using novel primers for the specific detection of *Pk*. This assay was able to detect 0.2 parasites/μL and presented a sensitivity of 96%, better than the other *Pk*-LAMP assay (Iseki et al., 2010) used for comparison. Nevertheless, the assay could not avoid the cross-reaction with *Pv*, resulting in a low species specificity (Britton et al., 2016).

As previously mentioned, molecular methods for malaria diagnosis, with LODs of 0.2-5 parasites/μL, are useful to detect submicroscopic infections. On the other hand, parasitemia in asymptomatic individuals and low-endemicity areas can be below those LODs. Mohon et al. (2019) reported the development of an ultrasensitive LAMP method capable of detecting 18S rRNA/DNA sequences from *Plasmodium* (Pan/*Pf*) from whole blood and dried blood spots, with LODs of 0.025-0.1 parasites/μL. Sensitivity of 97.0% and specificity of 99.1% were observed for the detection of all *Plasmodium* species in 494 samples from asymptomatic individuals (Mohon et al., 2019).

A recently published systematic review compared the diagnostic accuracy of LAMP methods for detecting malaria to the performance of microscopy and RDTs. The pooled sensitivity of LAMP ranged 96–98%, while specificity was ~95%, whichever the comparator (microscopy, PCR or RDT). Still, the study confirmed the higher difficulty of non-falciparum malaria diagnosis compared to *Pf* only (Picot et al., 2020). In the same way, a meta-analysis assessing the accuracy of LAMP tests as a diagnostic tool for uncomplicated malaria in endemic areas, showed the suitability of this method for detecting low-level malaria parasite infections. Nevertheless, the analysis also evidenced the focus of most studies on the diagnosis of Pan-Malaria or *Pf* only, while just 6/27 studies assessed *Pv* (Selvarajah et al., 2020). Other non-falciparum spp, as previously mentioned, remain even more neglected.

## Challenges and Perspectives

We believe that the main barrier to overcome in order to significantly advance in the specific diagnosis of non-falciparum malaria, is the development and transfer of knowledge among different diagnostic techniques, most of them mentioned in this review.

In addition to the challenge of analyzing and comparing reports using a wide variety of units (and even comparators) to express sensitivity, specificity, LOD and accuracy among approaches, the results obtained with some techniques are rarely exploited by others. For instance, most of the results showing promising or even excellent outcomes in the specific identification of *Plasmodium* spp by PCR-based techniques seem to be restricted to laboratorial analysis, with a few exceptions regarding lab-on-chip approaches. On the other hand, most potentially field applicable LAMP assays are based on targets and methods early described for LAMP implementation, rather than using recent and promising results obtained with PCR analyses. In the same way, typically biosensors for malaria use identical targets as RDTs, while seeking to improve sensitivity. The outcomes are promising for specific detection of *Pf* and, occasionally, *Pv*, while other non-falciparum spp remain neglected. The development of immunosensors is usually based on ELISA assays and the best malarial targets (antibodies) could be assayed. However, the general purpose of biosensing commonly is rapid detection rather than serological surveillance, and thus, this strategy was much less pursued.

In summary, the scientific community has the potential tools and extensive newly-generated knowledge at its disposal to create new, PoC and sensitive devices for the specific diagnosis of neglected non-falciparum spp. We look forward to seeing new diagnostic tools helping to control and eliminate malaria around the world.

## Author Contributions

AG and RM contributed to conception and design of the study. AG, RM, and MR performed investigation and data curation. AG, RM, and MR wrote the first draft of the manuscript. DB supervised the study and revised the manuscript. All authors contributed to the article and approved the submitted version.

## Funding

This study was supported by Serrapilheira Institute (Grant #G-1709-16618). AG is recipient of postdoctoral fellowship from Coordenação de Aperfeiçoamento de Pessoal de Ensino Superior (CAPES-PNPD).

## Conflict of Interest

The authors declare that the research was conducted in the absence of any commercial or financial relationships that could be construed as a potential conflict of interest.
